# Reinventing virtual care: Bridging the healthcare system and citizen silos to create an integrated future

**DOI:** 10.1177/08404704211062575

**Published:** 2022-04-26

**Authors:** Elizabeth M. Borycki, Andre W. Kushniruk

**Affiliations:** 1 8205 University of Victoria, Victoria, British Columbia, Canada.; 2 University of Victoria 8205 , Victoria, British Columbia, Canada.

## Abstract

The pandemic has accelerated the move to virtual care. This has included remote monitoring and implementation of technologies that allow for patient care at home and assisted living for ageing in place. Technologies are available to help consumers to maintain their health and wellness. However, challenges associated with implementing virtual care remain. In this article, we describe some of these challenges, along with the need to develop new models for promoting effective and sustainable virtual care. This includes the need for integration of institutional efforts (eg, government and hospital) with emerging access to commercially available home technologies supplied to patients and citizens. The authors argue that consideration of a personal digital ecosystem and its relation to institutional digital health ecosystems is critical. The authors suggest virtual care be considered in the combined context of the person and healthcare system. Implications for future research directions for virtual care are discussed.

## Introduction

Virtual care has emerged as an important trend that promises to transform healthcare, with the potential to make it more accessible, equitable, and economic. During the COVID-19 pandemic, virtual care emerged as an important and essential way of delivering healthcare. Virtual care has enabled the vulnerable to interact with healthcare providers and to access healthcare while at the same time avoiding unnecessary exposures to COVID-19 for patients as well as their healthcare providers. Virtual care has also allowed for patients to be remotely monitored at home rather than being admitted to hospital (in circumstances where this is clinically possible). Yet, even as virtual care has emerged as a key and critical mechanism to delivering healthcare across Canada and internationally, we still have a long way to go in terms of understanding the potential and policymaking surrounding the use of technologies to remotely monitor and support the healthcare needs of Canadians at home.

## Objectives

The objectives of this paper are three-fold: to define virtual care; to describe critical challenges that inhibit the implementation of virtual care; and to argue the need to develop virtual care within the context of the patient’s own digital ecosystem.

### Virtual care

Virtual care is a term that has been ascribed to the use of information and communication technologies used in healthcare. Virtual care has been defined as “any interaction between patients and/or members of their circle of care, occurring remotely, using any form of communication or information technologies with the aim of facilitating or maximizing the quality and effectiveness of patient care.”^1^ Internationally, virtual care has taken many forms – from monitoring individuals engaging in wellness activities (eg, exercise and diet) to telemedicine visits through to being a critical and recognized technology infrastructure used in the healthcare system to support individuals, who would normally be admitted to hospital to be cared for remotely. From a healthcare policy-making perspective, virtual care can be difficult to implement as it involves understanding:

1. Where a citizens’ digital health ecosystem begins and where it ends.
2. Understanding the effects of citizen choice surrounding their digital ecosystem and the ability of citizens to identify and access an evidence-base to support their choices surrounding those technologies that lead to positive behaviour change.
3. Its impacts on healthcare interventions, performance, and cost of care as a citizens’ health condition worsens or their physical and/or cognitive function changes.
4. The relation between public initiatives, for example, government and healthcare organizations (aimed at moving care from “bricks and mortar buildings” to remote care in the home) and private initiatives, for example, commercial efforts (aimed directly at promoting and supporting customer/citizen health, wellness, and independent living).

Policy-makers will need to address these issues as they are only beginning to understand the implications of a publically funded digital infrastructure that is pervasive and built around virtual care. In 2008, Niels Boye,^2^ a thought leader in the field of health informatics, identified that countries will not be able to sustain the costs associated with providing the technology infrastructure needed to effectively support healthcare services. Boye identified that there is a need to understand the citizens’ role and their technologies in the healthcare digital ecosystem and the healthcare process. He also noted that citizen use of technologies was expected to grow rapidly. At the time, access to the Internet, the use of mobile phones, and the growth of the mobile app sector pointed to a future, where citizens would supply some of their own personal technology infrastructure in the form of hardware and software to support their healthcare, and that this would be inevitable. In this future, citizens would choose technologies that fit with their lifestyles, underlying health conditions and their physical and cognitive needs to live happy healthy lives in the community. These citizens would in turn plug into the healthcare systems’ digital infrastructure and backbone, when experiencing healthcare events that required the support and management of health professionals. For example, digitally enabled citizens would maintain their own wellness and health-related technologies, until they required the help of health professionals during an acute medical event. These individuals would receive treatment in hospital and be discharged home for additional management and support that would leverage their existing personal digital ecosystem, which would be plugged into the institutional digital health ecosystem (with healthcare technology infrastructure dependence decreasing or ending over time as the individuals’ health and independence is restored).

Along these lines, Boye argued that new market models needed to be developed in order for virtual care “to pervade society and add value to the health aspects of an individuals’ life. Ethical and legal aspects must be further matured. Maturation of technology is needed.” In addition, it has become clear that new economic models that can support sustainable virtual care over time need to be developed. These models must consider and integrate contribution from both the public sector (eg, governmental and healthcare organizations) and the private sector (eg, vendors and service providers of technologies used in the home).

### The need for an integrated approach

The time for an integrated approach to digital health has arrived. COVID-19 has placed pressures on the healthcare system to advance virtual care, prevent citizen death, and protect the healthcare workforce from unnecessary exposures to the virus.^3,4^ In addition to this, climate change and the need to reduce our dependence on fossil fuels has increased our need to reduce travel associated with healthcare activities. Virtual care could lead to significant reductions in fossil fuel emissions and the costs associated with travel.^5^

Since 2008, we have seen a number of technological advancements in healthcare. The private sector has developed software and devices that can be easily used by healthcare consumers and their families. These technologies have improved in their quality, effectiveness, and usability. They have also become miniaturized and more ubiquitous and pervasive.^6^ Consumers and families are choosing what technologies to use and where to use them to support their health and wellness. Citizens and families are able to tailor these technologies to their needs and circumstances.^3^ The price of these technologies has dropped, for example, a mobile health app may have little to no cost associated with its use (eg, as little as a dollar for a health app). Medical devices that support self-monitoring have also come down in price and could be integrated with mobile apps downloadable from the platforms that service the majority of the North American population (ie, Google® Play, Apple® App Store, Windows® Store, and Amazon® Appstore).

To illustrate, citizen demand for oximeters increased during the height of the pandemic (as part of self-monitoring for low oxygen levels associated with COVID-19 infections). Citizens could buy oximeters on-line for less than $25 to more than $200 dollars that could integrate with their mobile phones. As the quality of these types of digital devices increase and the costs of private sector production continue to drop, newer and cheaper devices could be bought by consumers and swapped out for even cheaper and more effective and reliable devices to be plugged into the citizen’s personal digital health ecosystem. Researchers have found that citizens use mobile devices, mobile apps, and mobile digital health devices to support their healthcare and have created their own digital health ecosystems.^6-11^

**Figure 1.**
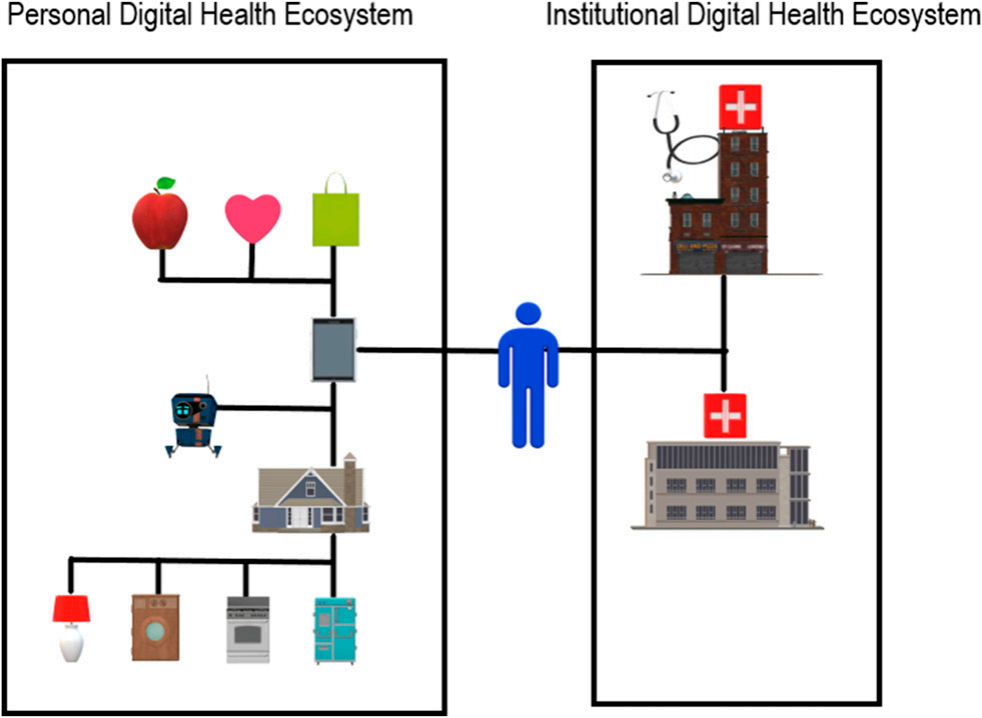
Personal digital health ecosystem and institutional digital health ecosystem silos.

Despite the potential positive and transformative impact of digital health, the systematic integration of these citizen purchased and personally controlled devices remains to be explored within the greater healthcare system. To date, the link between the formal healthcare technology infrastructure and the informal citizen controlled personal health technology ecosystems has remained to be fully explored and continues to remain siloed. Figure 1 illustrates this situation, with the citizen in the middle between their own evolving personal digital health ecosystem (that includes greater incorporation of smart home technologies, home computing, home robots, and smart phone devices) and the institutional digital health ecosystem (associated with their hospitals and physician offices or clinics).

The cost of publically funded, institutional health technology infrastructure continues to grow. Meanwhile, many consumers do not see the technologies they need to support their health in formal, publically funded organizations, so they continue to create their own personalized digital ecosystems that meet their own financial ability to pay for healthcare and support their wellbeing. To illustrate, in a recent publication by Dworak-Peck and colleagues, 94% of the homeless in Los Angeles owned a cell or mobile phone. Eighty six percent of the homeless owned a cell or mobile phone with an Android operating system, and 85% used their mobile phone daily for text messaging and Internet access.^7-9^ The research suggests a significant potential for tapping the digital ecosystems of the homeless population for accessing healthcare services and addressing equity and accessibility issues. Moreover, citizens from around the world are increasingly becoming accustomed to working with technologies and adopting consumer technologies that make their lives easier such as robotic vacuum cleaners and automated voice assistants. As citizens age, these technologies could help them to “age in place” taking over many of the activities that would prevent an individual from living in their home independently without some form of help, for example, a physical disability may prevent one from being able to vacuum one’s home and a robot could take over this activity.^10,11^

However, gaps remain. There is a need to assess the individual’s technological infrastructure (hardware, software and communication technologies) and training/support needs, as well as to stratify and document (where possible) the digital health needs of the population. At an individual level, health informatics professionals can assess what each individual already has available to access services (eg, mobile phone, access to a mobile app that helps with maintaining a regular exercise schedule, access to free Internet in the community, or paid Internet access in their home). Existing customized technology solutions could be used or created for individual citizens and those citizens that have similar health issues (eg, free, research-supported software apps that help with self-management of diabetes) and they could be made available through top technology platforms such as Google® Play, the Apple® App Store, the Windows® Store, and Amazon® Appstore that support the bulk of the consumer app market (ie, providing access to over 6.8 million apps).^12^ This needs to be integrated with the institutional digital health ecosystem (eg, recommended apps could be identified and uploaded to these platforms by regional health authorities for sanctioned use). Technological accessibility in remote and rural areas also remains an issue and will also need to be addressed. Alternate software and devices (that are not reliant on continuous, high-speed internet access) could be used in rural and remote areas where such access is not possible. Policy-makers may need to recognize the importance of such access in rural settings and develop commensurate strategies and funding support to ensure improved rural and remote access to digital services. Nordic countries such as Norway, Sweden, Finland, and Denmark have already done this as part of their digital health strategy.^13^

The integration of citizen digital ecosystems with formal institutional technology healthcare infrastructures remains a challenge and an untapped potential. Such integration would also have the potential to reduce costs for digital health and could form the basis for a more personalized, sustainable, and lower cost models for providing virtual healthcare. For this to happen, research is needed to understand what consumers already use, how they use these technologies, and how technology can be integrated within the formal healthcare system to provide tailored, seamless care and avoid costly duplication and inefficiencies. Such research will help us to document and understand how existing technology infrastructure is currently being used by citizens and how it is could be used to support health and wellness in the future. Citizens should be involved in identifying, documenting, and designing successful digital health approaches through participatory research methods.^14,15^ The development of a technology industry focused on consumer health hardware and software and the digitization and miniaturization of health technologies and devices has accelerated this need.

## Discussion and conclusion

Virtual care has emerged as an important method for delivering healthcare. Yet, many policy-making challenges exist when considering virtual care within the context of healthcare technology infrastructures. In designing and delivering virtual care resources health informatics and technology, administrators need to consider several aspects of the current healthcare environment including the following:

• The high cost associated with governments providing virtual care that does not leverage a citizen’s existing choices surrounding their own personalized digital ecosystem.
• The current evolved and mature state of consumer technologies that are being used by citizens to support their own health and wellness.
• The significant reductions in the cost of consumer health technologies that allow consumers to identify, select, and self-tailor their technologies to support their health and wellness.
• Consumer development of their own self-managed digital ecosystems.
• Consumer use of technologies in the workplace and the integration of new technologies in the home.
• The development of formal healthcare technology infrastructures as part of “bricks and mortar” healthcare systems that are separate from citizen digital health ecosystems.
• The existence of citizen health and wellness silos that are separate from healthcare institutional silos and models of care.
• The need for integration of the citizen and healthcare system digital infrastructures and policy to support the integration of technologies and models of care that allow for the promise of virtual care to manifest.

Policy-makers need to consider the current state of the virtual care environment and the silos that exist between citizen and healthcare organizations where health technology and data are concerned. Policy-makers must also understand that citizens choose technologies that support their personal health needs, and many of these technologies can supply citizens with the same health information and supports to enable their decision-making as healthcare institutions (eg, a citizen may buy an oximeter that interfaces with their cell phone or choose to use a smart watch to obtain the same information about their blood oxygen saturation). Such considerations are necessary to afford citizens with choice surrounding the use of technologies and to leverage citizens’ existing knowledge and personal digital health ecosystem when responding to a citizen’s identified health needs.^15,16^ In addition, a shift in cost and payment structures will need to occur as virtual care becomes more tightly embedded in the healthcare ecosystem, for example, policy-makers will need to develop payment schemes and fee schedules for physicians, home care agencies, and regional health authorities to provide virtual care and to avoid providing technologies and technology services that are already being used by citizens (eg, citizens who already use mobiles phones and tablets may not need to be provided with such technologies to access virtual care services by healthcare providers or healthcare organizations). Recognizing this allows society to avoid unnecessary costs and duplication of technologies and services.^17,18^ These considerations should be built into evidence-based procurement and technology selection processes for acquiring technologies that more effectively support virtual care.^19^

In Canada, data are being collected by Canada Health Infoway to assess citizen accessibility, overall need for virtual care, care needs based on rural or remote settings, and scope of virtual care in Canada, including during the pandemic.^20^ Along these lines, future research will need to assess virtual care success rates and examine how technologies can be integrated and interfaced so that varying models of care employed by healthcare institutions provide more integrated healthcare at a lower cost that is equitably accessed and tailored to citizens and their health needs.
